# Comparing Diabetes Mellitus Mortality Estimates from CDC WONDER Death Certificate Data and Global Burden of Disease Study Data in the United States

**DOI:** 10.34172/jrhs.12781

**Published:** 2026-02-21

**Authors:** Abdul Mannan Khan Minhas, Salim S. Virani, Harriette GC Van Spall, Dmitry Abramov

**Affiliations:** ^1^Section of Cardiology, Baylor College of Medicine, Houston, Texas, USA; ^2^Department of Medicine, Aga Khan University, Karachi, Pakistan; ^3^Department of Population Health, Aga Khan University, Nairobi, Kenya; ^4^Baylor College of Medicine Houston, TX, USA; ^5^Department of Medicine, McMaster University, Hamilton, Canada; ^6^Population Health Research Institute, Hamilton, Canada; ^7^Loma Linda University Medical Center Loma Linda, California, USA

**Keywords:** Diabetes, Mortality, Epidemiology

## Abstract

**Background:**

Data on mortality from Diabetes Mellitus (DM) in the United States (US) are available from various sources, including the Centers for Disease Control and Prevention Wide-Ranging Online Data for Epidemiologic Research (CDC WONDER) death certificate data and the Global Burden of Disease Study (GBD). This study aimed to compare DM mortality reporting between the CDC WONDER and GBD datasets.

**Methods:**

In this retrospective cohort study, we calculated the absolute number of deaths and crude mortality rates attributed to DM as the underlying cause of death in CDC WONDER and GBD in the US from 1999 to 2021 (CMR with 95% Confidence Intervals (CI) or Uncertainty Intervals (UI)) per 100000 population. Because of methodological differences between datasets, results were also evaluated based on the inclusion and exclusion of mortality from DM with renal complications.

**Results:**

The reported CMR for DM increased from 34 (95% CI: 34 to 35) to 41 (95% CI: 41to 42) in CDC WONDER but decreased from 36 (95% UI: 32 to 37) to 29 (95% UI: 27 to 31) in GBD from 1999 to 2021. When deaths from DM with renal complications were excluded from CDC WONDER to mirror GBD reporting, CMRs in 1999 and 2021 from CDC WONDER were 33 (95% CI: 33 to 34) and 29 (95% CI: 28 to 29), respectively, and trends were generally similar.

**Conclusion:**

Estimates of DM mortality rates and temporal trends in the US vary across commonly utilized sources of mortality data. These results have important implications for epidemiological efforts to understand and interpret DM mortality reporting.

## Background

 Accurate assessment of mortality attributed to Diabetes Mellitus (DM) in the United States (US) is important as DM is one of the leading causes of death.^1,2^ In the US, commonly utilized sources for disease-specific mortality include underlying or multiple cause of death files in the Centers for Disease Control and Prevention Wide-Ranging Online Data for Epidemiologic Research (CDC WONDER) and the Global Burden of Disease Study (GBD).^3,4^ Mortality data available from the CDC WONDER are derived from death certificate diagnoses which are subsequently coded as causes of mortality (based on the International Classification of Diseases [ICD] codes). Although data within the GBD are similarly based on ICD codes, the GBD data undergo further methodological adjustments and GBD mortality classifications may differ from other datasets.^5,6^ For example, CDC WONDER presents mortality for “insulin independent diabetes”, “insulin-dependent diabetes”, and “unspecified diabetes mellitus” while GBD reclassifies “unspecified” codes to either Type 1 or Type 2 DM. Additionally, codes for DM associated with renal complications may be included as mortality due to DM in CDC WONDER but are classified as mortality attributed to chronic kidney disease due to DM (reported in the chronic kidney disease mortality category) in GBD.^7^ Consequently, mortality estimates reported in CDC WONDER and GBD may differ in important ways, with prior analyses demonstrating notable differences between the datasets in mortality reporting for other causes of death.^8,9^ To further evaluate potential differences in DM mortality reporting, we evaluated mortality rates and mortality trends for DM in the US over the last two decades within the CDC WONDER and GBD datasets.

## Materials and Methods

 The data in this study were extracted from two publicly available datasets, the GBD 2021 (Global Burden of Disease Study 2021) and the underlying cause of death files in the CDC WONDER.^10,11^ The detailed methodology of estimation of mortality causes from the GBD has been previously described.^4^ GBD uses sophisticated methods to handle missing or incomplete data and updates methodology to improve statistical estimation over time. CDC WONDER presents data compiled from the National Center for Health Statistics, which contains over 99% of deaths in the US and represents complete counts without the risk of sampling error. Therefore, these data have been commonly used for mortality research as the definitive source for US mortality.^8,12^ DM was identified using ICD codes E10, E11, and E14 within CDC WONDER and was also separately evaluated excluding E1x.2 codes which describe DM with renal complications. Other DM codes available from CDC WONDER were not included as they yield a very small number of deaths yearly, generally under 50. Within GBD, we extracted DM mortality from the category labelled as DM, which does not include chronic kidney disease due to DM type 1 and chronic kidney disease due to DM type 2 (these two are given separately, i.e., in the chronic kidney disease category). Other than E1x.2, GBD classifies all other E1x codes, which include DM with other potential complications, as DM mortality. We also conducted a separate analysis in which mortality from DM with ICD codes E10, E11, and E14 from CDC WONDER was compared to GBD mortality from DM plus chronic kidney disease due to DM.

 To restrict the analyses to the adult population and to allow comparison of age ranges included in the datasets, we limited our cohort to a population aged ≥ 20 years. Crude mortality rates (CMR with 95% CI for CDC WONDER and 95% UI for GBD) per 100­000 population were extracted for DM and subcategories of DM by year. The denominator for data presented in GBD and CDC WONDER is based on the US population in the corresponding year. Percent change in DM deaths across years was calculated as ([deaths in 2021 – deaths in 1999]/[deaths in 1999] × 100). Average annual percent change (AAPC) using the Joinpoint Regression Program (National Cancer Institute) was also calculated with 95% CI, with confidence intervals not crossing 0 representing significant trends. Crude mortality rates are reported instead of age-standardized mortality rates to avoid the impact of different standard populations used for standardization.

## Results

 In 1999 and 2021 respectively, CDC WONDER reported a CMR (presented per 100­000 population) of 34 (95% CI: 34, 35) and 41 (95% CI: 41, 42) for DM, with CMR of 4 (95% CI: 4, 4) and 2 (95% CI: 2, 2) for insulin dependent DM, CMR of 5 (95% CI: 5, 5) and 17 (95% CI: 17, 17) for non-insulin dependent DM, and CMR of 25 (95% CI: 25, 25) and 23 (95% CI: 23, 23) for unspecified DM. In 1999 and 2021 respectively, GBD reported a CMR of 36 (95% UI: 32, 37) and 29 (95% UI: 27, 31) for DM, with a CMR of 2 (95% UI: 2, 2) and 1 (95% UI: 1, 1) for Type 1 DM and CMR of 34 (95% UI: 31, 35) and 28 (95% UI: 26, 30) for Type 2 DM. Comparing 1999 and 2021, overall DM mortality within CDC WONDER demonstrated an increase of 20.4% (AAPC 0.9%, 95% CI: 0.7, 1.1) while overall DM mortality within GBD demonstrated a decrease of 19.4% (AAPC -0.9%, 95% CI: -0.9, -0.8). CMRs for different subcategories of DM by year within CDC WONDER and GBD are presented in Table 1 and absolute mortality numbers are presented in Table 2.

**Table 1:** Crude Mortality Rates (CMR) for Diabetes Mellitus (DM) in CDC WONDER and GBD, with Percent Change between 1999 and 2021

	**GBD**			**CDC WONDER**				
	**DM**	**DM1**	**DM2**	**DM (E10, E11, E14)**	**Insulin dependent DM (E10)**	**Non-insulin dependent DM (E11)**	**Unspecified DM (E14)**	**DM (minus renal complications)**
**Year**	**CMR (95% UI)**	**CMR (95% UI)**	**CMR (95% UI)**	**CMR (95% CI)**	**CMR (95% CI)**	**CMR (95% CI)**	**CMR (95% CI)**	**CMR (95% CI)**
1999	36 (32, 37)	2 (2, 2)	34 (31, 35)	34 (34, 35)	4 (4, 4)	5 (5, 5)	25 (25, 25)	33 (33, 34)
2000	36 (33, 37)	2 (2, 2)	34 (31, 36)	34 (34, 35)	4 (4, 4)	6 (6, 6)	25 (25, 25)	34 (33, 34)
2001	37 (33, 38)	2 (2, 2)	35 (32, 36)	35 (35, 35)	3 (3, 4)	6 (6, 6)	25 (25, 25)	34 (34, 34)
2002	37 (34, 39)	2 (2, 2)	35 (32, 37)	35 (35, 36)	3 (3, 3)	7 (7, 7)	25 (25, 25)	35 (34, 35)
2003	37 (34, 39)	2 (2, 2)	35 (32, 37)	36 (35, 36)	3 (3, 3)	7 (7, 7)	25 (25, 26)	35 (34, 35)
2004	37 (33, 38)	2 (2, 2)	35 (32, 37)	35 (34, 35)	3 (3, 3)	7 (7, 7)	25 (24, 25)	34 (34, 34)
2005	37 (33, 39)	2 (2, 2)	35 (32, 37)	35 (35, 35)	3 (2, 3)	8 (8, 8)	25 (25, 25)	34 (34, 35)
2006	36 (33, 38)	2 (2, 2)	34 (31, 36)	34 (33, 34)	2 (2, 2)	8 (8, 8)	24 (23, 24)	32 (32, 33)
2007	35 (31, 36)	2 (2, 2)	33 (30, 35)	33 (32, 33)	2 (2, 2)	8 (8, 8)	23 (23, 23)	31 (31, 31)
2008	34 (31, 36)	2 (1, 2)	32 (29, 34)	32 (32, 32)	2 (2, 2)	8 (8, 8)	22 (22, 22)	30 (30, 31)
2009	33 (29, 34)	1 (1, 2)	31 (28, 33)	31 (30, 31)	2 (2, 2)	8 (8, 8)	21 (21, 21)	29 (29, 29)
2010	31 (28, 33)	1 (1, 1)	30 (27, 31)	31 (30, 31)	2 (2, 2)	8 (8, 8)	21 (21, 21)	29 (28, 29)
2011	29 (26, 30)	1 (1, 1)	28 (25, 29)	32 (32, 32)	2 (2, 2)	8 (8, 8)	22 (22, 22)	25 (24, 25)
2012	28 (25, 29)	1 (1, 1)	27 (24, 28)	32 (32, 32)	2 (2, 2)	8 (8, 8)	22 (22, 22)	24 (24, 25)
2013	27 (24, 28)	1 (1, 1)	25 (23, 27)	32 (32, 33)	2 (2, 2)	9 (8, 9)	22 (22, 22)	23 (23, 23)
2014	27 (24, 28)	1 (1, 1)	25 (23, 27)	32 (32, 33)	2 (2, 2)	9 (9, 9)	22 (22, 22)	23 (23, 23)
2015	27 (24, 28)	1 (1, 1)	26 (23, 27)	33 (33, 33)	2 (1, 2)	10 (10,10)	22 (22, 22)	23 (23, 24)
2016	27 (25, 29)	1 (1, 1)	26 (24, 28)	33 (33, 33)	2 (1, 2)	11 (11, 11)	21 (21, 21)	23 (23, 23)
2017	28 (25, 29)	1 (1, 1)	27 (24, 28)	34 (34, 35)	2 (2, 2)	12 (12, 12)	21 (20, 21)	24 (24, 24)
2018	28 (26, 30)	1 (1, 1)	27 (24, 28)	35 (34, 35)	2 (1, 2)	13 (13, 13)	20 (20, 21)	24 (24, 24)
2019	29 (26, 30)	1 (1, 1)	27 (25, 29)	36 (35, 36)	2 (1, 2)	14 (13, 14)	20 (20, 21)	25 (25, 25)
2020	29 (26, 30)	1 (1, 1)	28 (25, 29)	41 (41, 41)	2 (2, 2)	16 (16, 16)	23 (23, 23)	29 (29, 29)
2021	29 (27, 31)	1 (1, 1)	28 (26, 30)	41 (41, 42)	2 (2, 2)	17 (17, 17)	23 (23, 23)	29 (28, 29)
Percent Change	-19.4%	-26.0%	-16.7%	20.4%	-60.5%	229.4%	-8.8%	-13.6%
AAPC	-0.9 (-0.9, -0.8)	-1.3 (-1.4, -1.2)	-0.8 (-0.9, -0.8)	0.9 (0.7, 1.1)	-4.1 (-4.3, -3.9)	5.7 (5.4, 5.9)	-0.4 (-0.6, -0.2)	-0.6 (-0.9, -0.3)

UI: Uncertainty Interval; CI: Confidence Interval; AAPC: Average Annual Percent Change

**Table 2:** Absolute Numbers of Deaths from DM in CDC WONDER and GBD, with Percent Change between 1999 and 2021

	**GBD**			**CDC WONDER**				
	**DM**	**DM1**	**DM2**	**DM (E10, E11, E14)**	**Insulin dependent DM (E10)**	**Non-insulin dependent DM (E11)**	**Unspecified DM (E14)**	**DM without renal complications**
**Year**	**Deaths (95% UI)**	**Deaths ((95% UI)**	**Deaths ((95% UI)**	**Deaths**	**Deaths**	**Deaths**	**Deaths**	**Deaths**
1999	70208 (63981, 73330)	3415 (3328, 3470)	66793 (60636, 69861)	68306	8478	10093	49714	66556
2000	71794 (65526, 74993)	3526 (3434, 3584)	68268 (62068, 71416)	69222	7746	11341	50119	67549
2001	74040 (67667, 77489)	3601 (3511, 3658)	70439 (64139, 73832)	71278	7100	12832	51334	69580
2002	76054 (69525, 79605)	3700 (3602, 3762)	72354 (65910, 75857)	73159	6813	14199	52136	71453
2003	77166 (70451, 80793)	3680 (3581, 3744)	73486 (66849, 77091)	74135	6227	14946	52945	72407
2004	76746 (69959, 80467)	3593 (3499, 3664)	73154 (66435, 76863)	73050	5763	15479	51795	71310
2005	77909 (70938, 81659)	3588 (3496, 3654)	74321 (67406, 78053)	75023	5469	16620	52911	73180
2006	76774 (69813, 80603)	3550 (3459, 3623)	73224 (66321, 77006)	72373	5073	16491	50790	69918
2007	75174 (68187, 79058)	3438 (3338, 3518)	71737 (64833, 75539)	71299	4704	16976	49608	68129
2008	74157 (67082, 78065)	3344 (3254, 3413)	70813 (63824, 74674)	70468	4445	17538	48469	66925
2009	72539 (65614, 76509)	3264 (3174, 3328)	69275 (62432, 73184)	68641	4157	17455	47014	64649
2010	70088 (63313, 73920)	3148 (3061, 3214)	66940 (60242, 70729)	69012	4075	17707	47204	64284
2011	65850 (59524, 69494)	3038 (2954, 3099)	62812 (56554, 66420)	73767	4141	19084	50497	56374
2012	64405 (58047, 67916)	3001 (2911, 3064)	61404 (55100, 64878)	73863	3767	19270	50788	56547
2013	62431 (56277, 65803)	2976 (2886, 3037)	59455 (53343, 62805)	75514	3801	20139	51522	53099
2014	62945 (56768, 66394)	2996 (2907, 3061)	59949 (53815, 63341)	76453	3684	20743	51961	53909
2015	64498 (58283, 67989)	3067 (2977, 3122)	61431 (55261, 64883)	79472	3610	23124	52674	55867
2016	66127 (59958, 69629)	3148 (3055, 3206)	62980 (56857, 66463)	79989	3677	26102	50159	55943
2017	67922 (61671, 71542)	3199 (3106, 3258)	64723 (58531, 68340)	83472	3881	29212	50314	58217
2018	69144 (62725, 72898)	3213 (3129, 3271)	65931 (59573, 69636)	84844	3727	30984	50089	59393
2019	71107 (64470, 74925)	3249 (3153, 3320)	67858 (61287, 71632)	87572	3734	33326	50450	61071
2020	72039 (65148, 76056)	3240 (3123, 3323)	68799 (61990, 72802)	102057	4288	40290	57407	71722
2021	73927 (67307, 78340)	3223 (3111, 3321)	70703 (64148, 75046)	103153	4230	41905	56922	72619
Percent Change	5.3%	-5.6%	5.9%	51.0%	-50.1%	315.2%	14.5%	9.1%

UI: Uncertainty Interval; AAPC: Average Annual Percent Change

**Figure 1:**
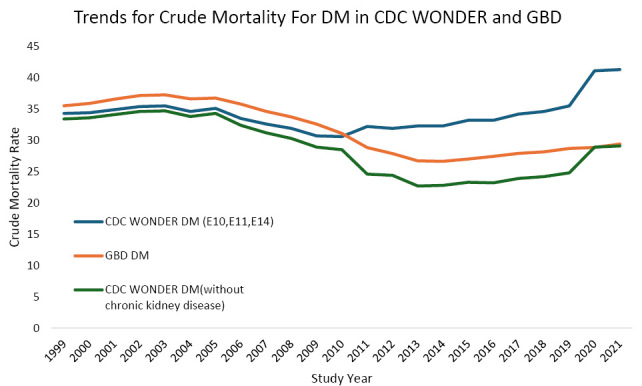
Trends in Crude Mortality Rate (CMR) for DM in CDC WONDER and GBD

 Mortality trends from CDC WONDER and GBD are displayed in Figure 1. Crude DM mortality rates from CDC WONDER and DM from GBD paralleled each other from 1999 to 2010. The CMRs were noted to cross in 2010 and subsequently diverge until 2019. CDC WONDER DM CMRs were noted to rise after 2010 while GBD DM CMRs initially fell after 2010, with GBD and appropriately matched CDC WONDER CMRs (without renal disease-related deaths) overlapping in the last two years of the study.

 When CDC WONDER data for DM mortality were evaluated after excluding ICD-10 codes for DM with renal complications, the CMRs for 1999 and 2021 were 33 (95% CI: 33 to 34) and 29 (95% CI: 28 to 29), respectively, with a decrease of 13.6%. These data demonstrate remaining differences in CMRs, with similar CMR trends during the study period (20.5% increase in the CDC WONDER, 23.3% increase in the GBD).

## Discussion

 This analysis of mortality attributed to DM from two commonly utilized datasets, CDC WONDER and GBD, demonstrates several important findings. Variation in methodological approaches between the datasets leads to differences in reported DM mortality rates and trends. In other words, DM mortality rates have been increasing over the last two decades based on CDC WONDER data but the rates have been decreasing based on GBD data. However, when DM with renal complications is evaluated similarly between the datasets (either included or excluded from both), the DM mortality trends in CDC WONDER and GBD are generally aligned. Appreciation of the variations in DM mortality reporting between common sources has important implications for epidemiologic estimates of the burden of DM mortality in the US, especially when findings appear to be discordant.

 Methodological differences in mortality reporting between CDC WONDER and GBD stem from several factors. CDC WONDER data on DM mortality, with the inclusion of ICD-10 codes E10, E11, and E14, include DM associated with other complications, such as DM with renal complications (E1x.2 codes). On the other hand, categorization of DM mortality in the GBD does not include mortality attributed to DM with renal complications, as the DM with renal complications is classified within the chronic kidney disease category. To bridge differences in reporting, excluding DM with renal complications from overall DM mortality data from the CDC WONDER database or adding chronic kidney disease due to DM to DM mortality data from the GBD allows for more comparable estimates of DM mortality reporting between datasets.

 Additional differences between datasets, which may result in differences in mortality reporting, are related to the classification and re-classification of DM mortality codes. CDC WONDER includes mortality data for unspecified diagnoses, such as “unspecified diabetes mellitus”, while GBD applies statistical modification to redistribute unspecified DM to either Type 1 or Type 2 DM based on patient age and other factors.^5^ Redistribution of mortality codes from nonspecific causes of death, termed “garbage codes” by the GBD, may also lead to deaths not initially coded as DM mortality to be redistributed to DM mortality.^6^

 Deaths ultimately attributed to DM in GBD may initially be coded as various conditions deemed ill-defined by GBD, including sepsis, heart failure, shock, osteomyelitis, and others.^6^ These types of methodological differences have been previously shown to cause variations in mortality estimates for other important causes of mortality, including accidents and cardiovascular conditions.^8,9^ For example, prior analyses comparing GBD and CDC WONDER identified notable differences in reported trends of cardiovascular disease mortality and also highlighted that the reported mortality attributed to ischemic heart disease was approximately 50% higher in GBD than in CDC WONDER in 2019.^12^ The findings from prior comparative studies between GBD and CDC WONDER as well as our findings regarding differences in DM mortality reporting have potentially important public health implications. For example, public funding and public health efforts focusing on prevention, screening, and treatment for particular conditions may be prioritized based on mortality trends. Therefore, it is important to consider methodological differences in mortality estimates across datasets when reporting and interpreting death data, including common conditions such as DM, as differences may affect not only research findings but also public health priorities. For completeness, evaluation of mortality from multiple datasets may also be considered.

 Although our analysis only evaluated the underlying cause of mortality from CDC WONDER to facilitate comparison with GBD data, CDC WONDER may also be queried for contributing causes of mortality. Utilizing both underlying and contributing causes provides a greater estimate for the contribution of a particular condition to mortality and may be more comprehensive than relying on underlying cause alone.^13^ Evaluation of multiple causes of mortality on one death certificate, as possible within CDC WONDER but not GBD, may be particularly important for future analyses that examine conditions with shared risk factors and closely linked clinical conditions such as DM and chronic kidney disease.

 Our findings have limitations. Determining the cause of mortality relies on accurate diagnoses and coding at the time of death. Coding practices in the United States have changed over time to align with quality and billing metrics, which may be reflected in greater utilization of more specific Type 1 and Type 2 DM codes versus unspecified codes and greater utilization of chronic kidney disease codes.^14,15^ Therefore, differences in coding over time, particularly with an increased focus on diagnosis and coding for chronic kidney disease, may explain some of the differences in mortality trends between the CDC WONDER and GBD databases in the second half of the study. GBD methodologies were developed to allow worldwide comparisons, and it is possible that the broad application of statistical modeling for countries with high-quality mortality data may require further evaluation. The final years of the study (2020-2021) overlap with the early phase of the COVID pandemic, which has been shown to affect trends of mortality from conditions such as DM.^2^ Further pandemic-related analyses are not specifically reported in the current study.

**Highlights**

- Various sources for mortality data in the United States are available.
- There are differences in Diabetes Mellitus mortality reporting between datasets.
- Differences in mortality reporting between datasets have important implications.

## Conclusion

 In conclusion, we highlight differences in DM mortality reporting between commonly utilized sources for mortality data in the US. These results have important implications for epidemiological efforts to understand and interpret mortality reporting for common causes of mortality, such as mortality due to DM.

## Acknowledgments

 None.

## Artificial Intelligence Use Disclosure

 No artificial intelligence was used for writing this manuscript.

## Competing Interests

 None.

## Ethical Approval

 Approval from an Institutional Review Board and informed consent were not necessary as the study utilized publicly accessible de-identified data.

## Funding

 This study was not financially supported by any public or private organizations.
